# Dynamics of Adatom and Vacancy Islands on Au(111) in Alkaline and Acidic Media

**DOI:** 10.1021/acs.jpcc.5c03661

**Published:** 2025-08-01

**Authors:** Toni Moser, Francesc Valls Mascaró, Julia Kunze-Liebhäuser

**Affiliations:** 27255 University of Innsbruck, Innrain 52c, Innsbruck 6020, Austria

## Abstract

Gold (Au), especially single-crystalline Au(111), has been extensively studied in fundamental electrochemistry due to its unique properties, including high conductivity, chemical stability, and well-defined surface characteristics, which make it an ideal model system for electrochemical investigations. At the same time, the Au(111) surface is known to degrade during oxidation–reduction cycling in commonly used electrolytes, exhibiting the formation of atomic-scale vacancy and adatom islands. Although there are many fundamental studies on these degradation processes, only a limited number of studies have been performed in alkaline media. In this work, we study the roughening and healing characteristics of Au(111) upon oxidation–reduction treatments in H_2_SO_4_ and NaOH. We demonstrate that the surfaces are less rough after treatments in NaOH due to rapid smoothening over time. These observations point toward a higher driving force for island decay and to a higher surface mobility in NaOH, which substantially accelerates the surface healing process.

## Graphical abstract

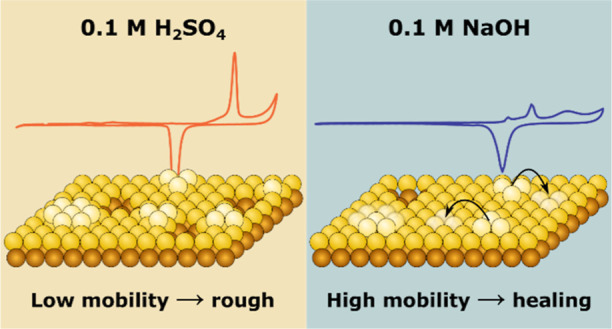

## Introduction

The stability of electrode materials is one of the most essential parameters when it comes to application in energy conversion technologies. It is determined by processes such as surface reordering, dissolution, and redeposition during anodic and cathodic polarization. Electrode materials made of gold (Au) and its alloys are regularly utilized. Due to its chemical inertness, an almost ideal polarizability compared to other noble metals, and its well-known surface structure, single-crystalline Au(111) is frequently used as a standard for a variety of electrochemical reactions and has been subject of fundamental research for many decades. This makes Au(111) one of the most important model systems for fundamental investigations in electrochemistry.^1,2^

Dissolution is a temporary process that takes place during both anodic oxidation and cathodic reduction. While dissolution is often rather moderate in anodic direction, it is usually more significant during reduction.^3^ There is a large number of fundamental studies on Au(111) oxidation–reduction cycling (ORC). Most of them have, however, been conducted in acidic electrolytes.^4–21^ The Au(111) surface undergoes morphological degradation during ORC in acidic media. This morphological evolution results in increased surface roughness and is strongly influenced by the potential range, scan rate, and electrolyte purity.^7,18,21^ Electrochemical scanning tunneling microscopy (EC-STM) investigations in acid show that when the reduction of the surface oxide is conducted via a potential step, a large number of both adatom islands and holes are formed, while only holes form if the reduction is performed via a linear sweep.^18^ The surface roughens more with repeated cycling. However, there is ongoing uncertainty in the literature regarding whether the surface can heal when cycling is halted and the potential is held within the double-layer region.^11,18,20^

Given that Au is the single metal catalyst with the highest activity toward carbon monoxide (CO) electro-oxidation in alkaline electrolyte,^22^ and considering the increasing importance of alkaline electrolyzer and fuel cell technologies,^23^ there is an urgent need to focus fundamental research more closely on oxidation and reduction phenomena in alkaline environments. This is particularly important given that the dissolution of polycrystalline Au during ORC in alkaline media is more than an order of magnitude higher than in acidic media.^3^

In this study, the morphology evolution of Au(111) in 0.1 M NaOH versus 0.1 M H_2_SO_4_ after oxidation and reduction treatments is closely investigated. The surface mobility is significantly higher in 0.1 M NaOH compared to that observed in 0.1 M H_2_SO_4_. This high mobility enables a very fast “healing” of the surface in the alkaline electrolyte, even after 3D roughening and visible disintegration of the (111) surface upon Au^3+^ oxide formation.

## Methods

### Au­(111) Preparation

The Au(111) single crystal (MaTecK, Ø = 10 mm, 99.999% purity, and polished <0.1°) was cleaned in a freshly prepared Caro acid solution and subsequently rinsed and boiled at least three times in ultrapure water (Milli-Q, Merck, 18.2 MΩ cm). Subsequently, the crystal was flame annealed in a propane flame to orange glow for 1 min and cooled down in an Ar (Messer, 99.999% purity) flow.

### EC-STM Studies

STM tips were prepared by electrochemical etching of a Pt_80_Ir_20_ wire (Goodfellow, Ø = 0.25 mm) in a 4 M KSCN/2 M KOH solution. The tips were subsequently coated with Apiezon wax. EC-STM measurements were performed using a Bruker Multimode 8 Scanning Tunneling Microscope placed inside an Ar-filled glovebox (MBraun MB 200 MOD). This ensures that the presence of oxygen inside the glovebox never exceeds 5 ppm. The prepared 0.1 M NaOH (Merck, 99.99% trace metal basis) and 0.1 M H_2_SO_4_ (Merk, 96% suprapur) solutions were purged with Ar to remove the dissolved oxygen.

The Au(111) crystal was placed inside a custom EC-STM cell made of polychlorotrifluoroethylene (PCTFE), which was cleaned in Caro acid and thoroughly rinsed and boiled in ultrapure water. Polytetrafluoroethylene (PTFE) bound activated carbon was used as both quasi-reference and counter electrodes.^24^ All recorded potentials are converted to the reversible hydrogen electrode (RHE) scale for better comparison with literature values.

Prior to all experiments, quality and cleanliness of the surface were routinely checked by STM imaging of the $\sqrt{3}$ × 22 (Herringbone) reconstructed Au(111). Gwyddion was used for data representation and for analysis of the STM images.^25^

## Results and Discussion

Figure 1 shows the cyclic voltammograms (CVs) of Au(111) in acidic and in alkaline electrolytes. In 0.1 M H_2_SO_4_, the low currents at potentials below 0.5 V_RHE_, in the double layer region, are attributed to the charging and discharging of the double layer at the Herringbone (HB) reconstructed surface.^26,27^ The reconstruction lifts due to the adsorption of sulfate anions as the potential is increased to 0.65 V_RHE_.^16,26^

**Figure 1:**
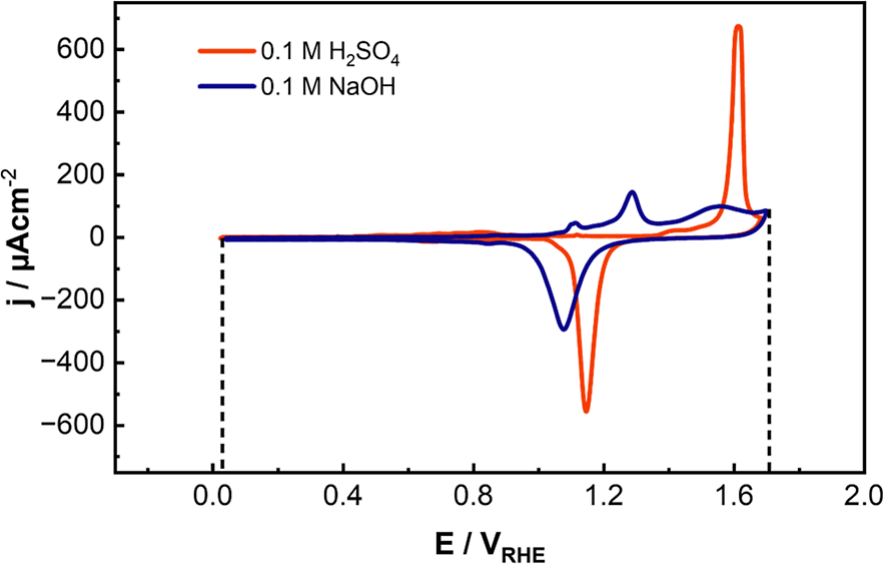
Cyclic voltammograms (CVs) of Au(111) measured in different electrolytes: 0.1 M H_2_SO_4_ (orange) and 0.1 M NaOH (blue). Both CVs were recorded in the EC-STM cell at 50 mV/s. The dashed black lines indicate the potentials applied during the oxidation–reduction treatment that results in surface roughening. At the upper vertex potential of 1.7 V, the oxidation charge density is very similar for both electrolytes, 897 μC cm^–2^ in 0.1 M H_2_SO_4_ and 868 μC cm^–2^ in 0.1 M NaOH, suggesting that the electrode surface reaches the same oxidation state in both media.

The oxidation of the gold surface initiates through OH adsorption at the (defect) step sites, resulting in the broad peak that starts appearing at 1.45 V_RHE_.^11,28^ This is followed by oxidation of the terraces at 1.6 V_RHE_, involving OH dehydrogenation into adsorbed O and a place-exchange of oxygen atoms with the gold surface atoms, which results in subsurface oxygen formation.^1,12,16,17,28–30^ The anodic oxide is readily reduced during the reverse cathodic scan, which gives rise to the sharp cathodic peak at 1.15 V_RHE_.

In 0.1 M NaOH, the lifting of the Herringbone reconstruction is induced by the adsorption of OH, which takes place at 1.10 V_RHE_.^15,31^ At higher anodic potentials, terrace oxidation starts and causes an anodic peak at around 1.30 V_RHE_, i.e., 0.3 V less positive than in 0.1 M H_2_SO_4_, where the surface is blocked by adsorbed sulfate anions which prevent an early oxidation.^15,31–33^ The third broad anodic peak at around 1.5 V_RHE_ is attributed to further oxidation of the terraces and the formation of Au^+^ species, as confirmed by electrochemical (near-ambient pressure) X-ray photoelectron spectroscopy (EC-XPS).^34^

The morphological evolution of the surface upon oxidation and reduction is tracked in situ with EC-STM. Figure 2 shows EC-STM images of the Au(111) surface after anodic polarization at 1.7 V_RHE_ and subsequent reduction at 0.05 V_RHE_. In 0.1 M H_2_SO_4_, this treatment results in the formation of adatom islands with monatomic height and vacancy islands with monatomic depth (see Figure 2a and the respective cross sections in the Supporting Information Figure S1), as it was already observed in previous studies.^5,7,9–11,14,18,20,35,36^

**Figure 2:**
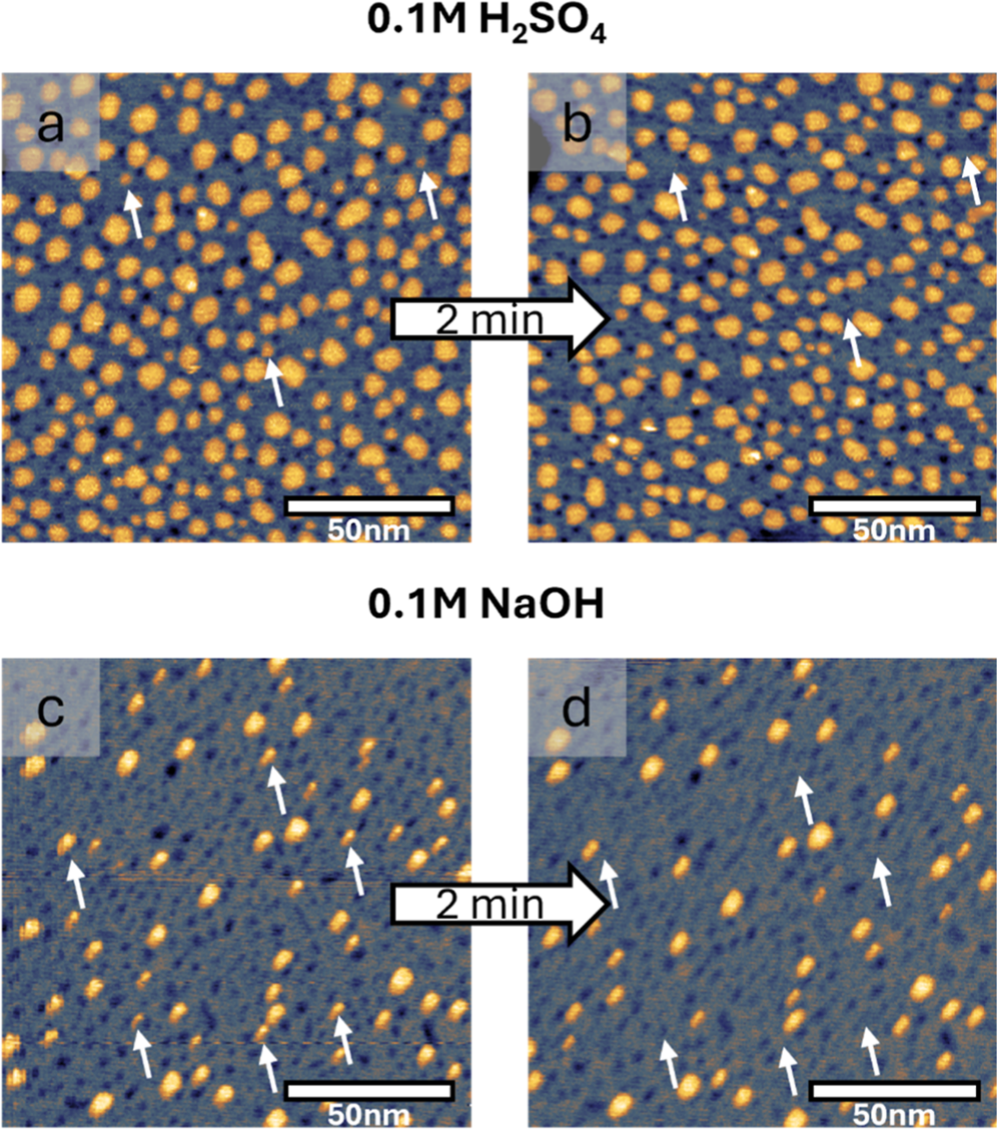
EC-STM images of Au(111) with adatom and vacancy islands formed during oxidation–reduction treatments. The treatment involves anodic polarization at 1.7 V_RHE_ for 1 min, followed by reduction at 0.05 V_RHE_. Top images: Au(111) in 0.1 M H_2_SO_4_ (a) directly after reduction at 0.05 V_RHE_ and (b) after 2 min at 0.05 V_RHE_. Bottom images: Au(111) in 0.1 M NaOH (c) directly after reduction at 0.05 V_RHE_ and (d) after 2 min at 0.05 V_RHE_. The white arrows point at islands that shrink or disappear within 2 min of reduction time. All image sizes are 140 × 140 nm^2^. *I*_tip_ = 1 nA, *E*_tip_ = 0.2 V_RHE_.

The islands originate from the nucleation of adatoms pushed out onto the terrace during the place-exchange that takes place at oxidative potentials, while the vacancy islands are mainly formed during the reduction of the surface oxide.^37,38^ In addition, part of the lifted gold atoms dissolve into the electrolyte and later redeposit onto the surface when the oxide is reduced, so that they contribute to additional island formation.^3,18^ Notably, the vacancy islands are substantially smaller than the adatom islands, which is an indication for the lower mobility of the vacancies, i.e., their shorter diffusion lengths lead to smaller vacancy island sizes and higher vacancy island densities.^39,40^ This apparent size difference might be slightly overrated due to tip convolution effects, which can lead to an overestimation of the adatom island and an underestimation of the vacancy island sizes. In the consecutive image shown in Figure 2b, measured 2 min later, only minor changes are observed in acidic media. Specifically, only few smaller islands disappear over time, as indicated by the white arrows. Figure 2c shows an EC-STM image of the surface after anodic polarization at 1.7 V_RHE_ and subsequent reduction at 0.05 V_RHE_ in 0.1 M NaOH. In analogy to the observations made in acidic media, the oxidation–reduction treatment results in the formation of monoatomically high adatom islands and vacancy islands. However, the density of islands formed during oxidation is smaller in alkaline media. Additionally, it decreases more rapidly upon stepping the potential to 0.05 V_RHE_ and holding it for 2 min (see Figure 2c,d). This is a clear indication of the lower stability of the islands, either due to a higher driving force to reestablish the flat Au(111) terraces, in case of a higher free energy of step formation in NaOH than in H_2_SO_4_, and/or due to an enhanced tendency for adatom diffusion in alkaline media.

In order to quantify the morphological evolution of the roughened Au(111) surface with time, we measured the areas of the adatom and vacancy islands in all four EC-STM images shown in Figure 2. For this analysis, we marked all features that appear 100 pm above the original, pristine terrace level as islands and those that appear 50 pm lower as vacancies. We excluded one-pixel large islands and vacancies from the quantification since they are likely due to noise. The masked images are shown in Figures S2 and S3, while the results from the quantification are plotted as histograms in Figure 3.

**Figure 3:**
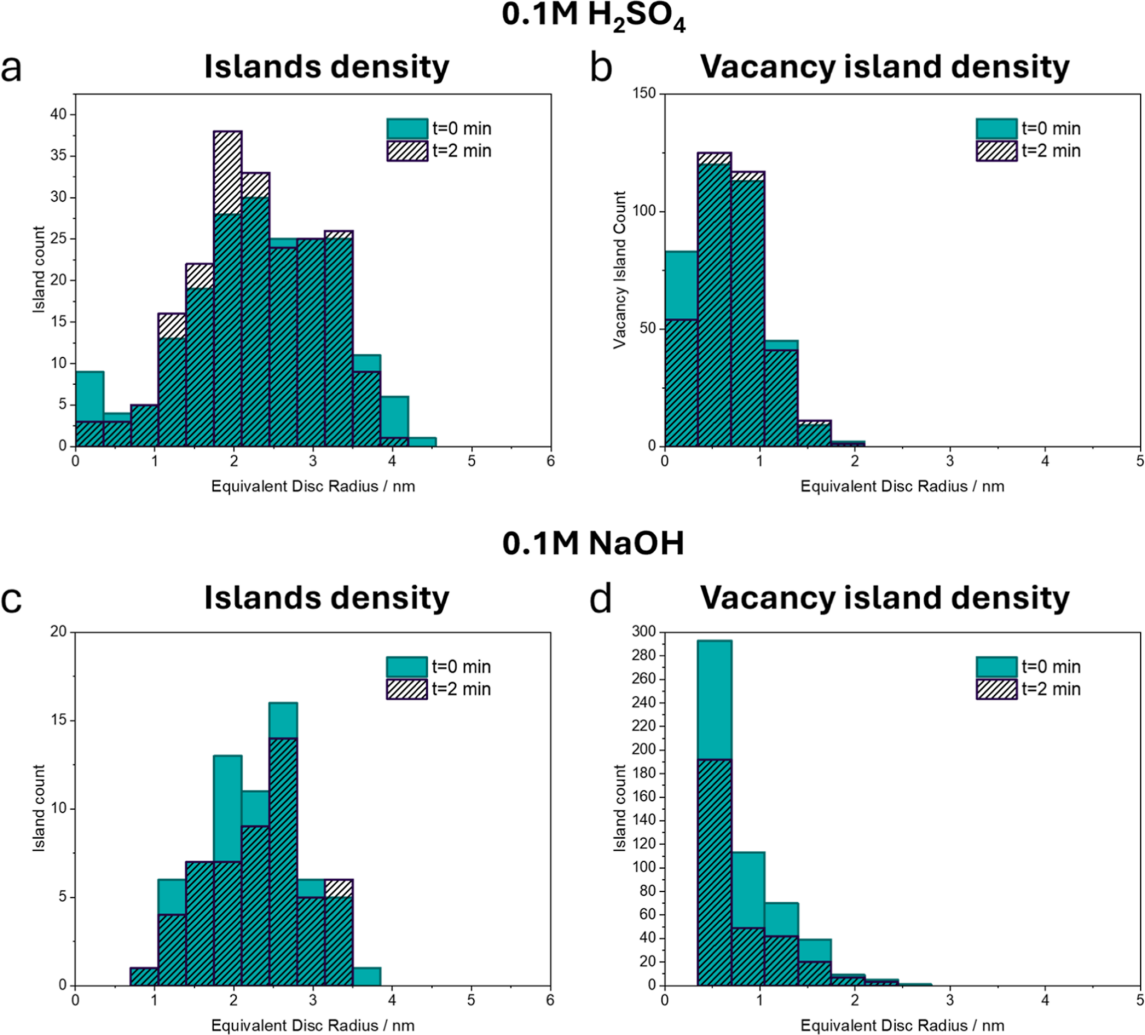
Size distribution of adatom and vacancy islands. Quantification of adatom and vacancy island sizes and densities from Figure 2 and their evolution with time at 0.05 V_RHE_, in 0.1 M H_2_SO_4_ (a,b) and in 0.1 M NaOH (c,d). The number of adatom islands (a,c) and vacancy islands (b,d) is plotted versus their equivalent disc radius for the first (Figure 2a,c) (turquoise-filled bars) images, recorded immediately, and the second (Figure 2b,d) (gray-striped bars) images, recorded 2 min after oxide reduction.

Figure 3 shows the size distributions of adatom and vacancy islands in acidic and alkaline media, immediately after reduction of the surface oxide (turquoise filled bars) and after 2 min at 0.05 V_RHE_ (gray striped bars).

In acid (Figure 3a,b), only the smallest adatom and vacancy islands are mobile and disappear, likely by Ostwald ripening, i.e., through atom-by-atom diffusion from the smaller to the larger islands, and/or by Smoluchowski ripening, i.e., through diffusion of small islands and aggregation into larger ones.^41–43^ The total area of adatom islands (i.e., the sum of all adatom island areas) decreases by approximately 5%, while there is almost no decrease (less than 1%) of the total area of vacancy islands (see Table S1 in the Supporting Information).

In alkaline media (Figure 3c,d), the reduction time at 0.05 V_RHE_ has a significant effect on the number and size distribution of the adatom and vacancy islands. While the number of large adatom islands (>3 nm radius) stays almost constant, the number of islands with intermediate size (1.5 nm −3 nm radius) decreases significantly. It is important to note that an accurate quantification of the area of adatom and vacancy islands is nontrivial due to tip–sample convolution effects in STM imaging, which tend to cause an overestimation of adatom island radii and an underestimation of vacancy island radii.^45^ In any case, the decrease in total area of adatom islands (see Table S2) cannot be explained solely by Ostwald and Smoluchowski ripening. As we also observe a general decrease in the number and area of vacancy islands (Figure 3d), it is very likely that in 0.1 M NaOH adatoms can surpass the Ehrlich–Schwoebel (ES) barrier for downward interlayer diffusion and hop down into vacancy islands, (partially) filling them.^44,45^ In addition there could be an effect from redeposition of gold atoms that were dissolved during the oxidation reduction treatment. Gold dissolution within the double layer region can be ruled out through earlier ICP–MS studies.^3^

In order to further study the interlayer diffusion of the adatoms in both acid and alkaline media, we produced larger, 3-dimensional (3D) islands by repeatedly oxidizing and reducing the Au(111) surface 10 times. In each cycle we applied 1.7 V_RHE_ for 10 s and then reduced the formed oxide by holding the potential at 0.05 V_RHE_ for 10 s. EC-STM images at 0.05 V_RHE_, recorded directly (Figure 4a,c) and 4 min after this treatment (Figure 4b,d), depict very different surface morphologies of Au(111) in acid (Figure 4a,b) and in alkaline (Figure 4c,d) media (additional images are shown in Figure S4).

**Figure 4:**
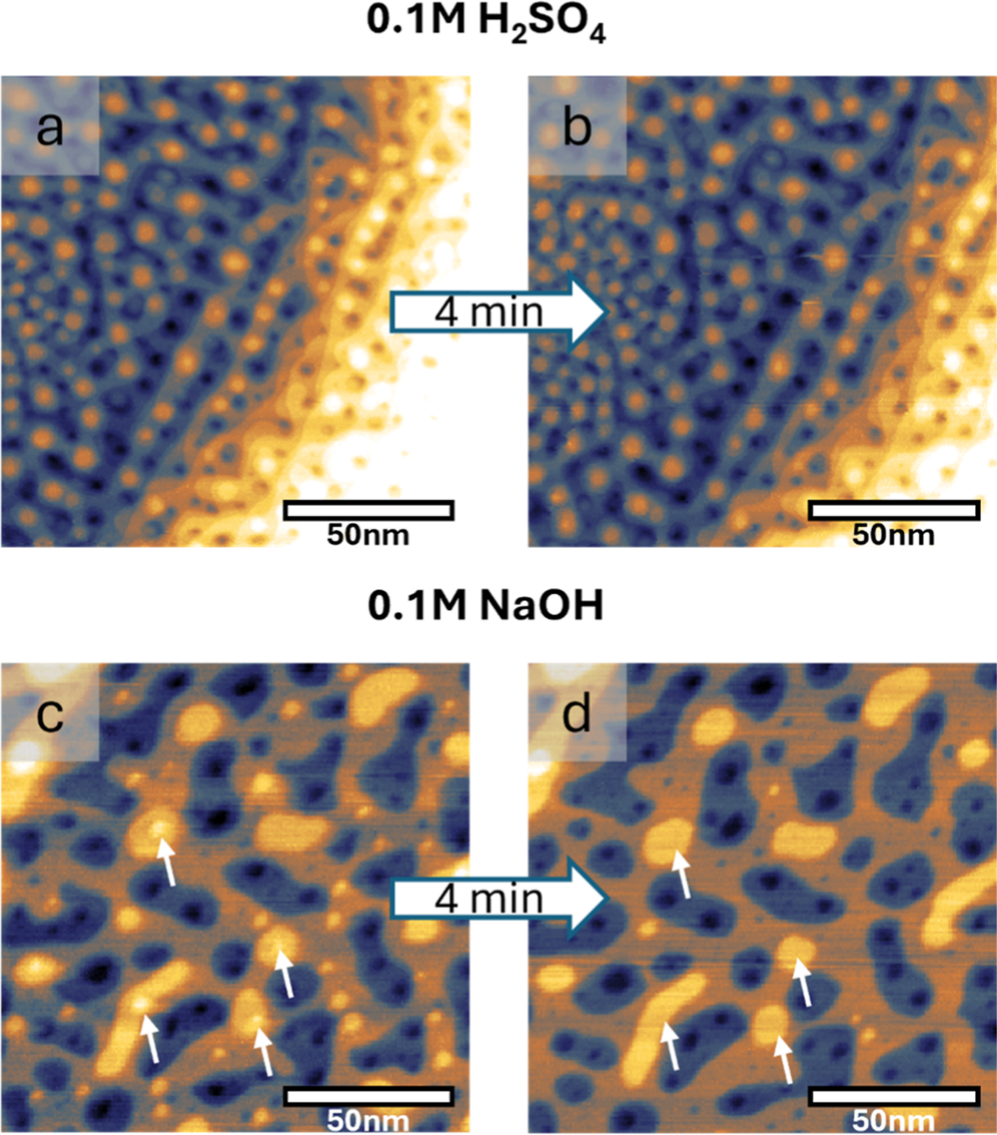
EC-STM images of Au(111) after 10 oxidation–reduction treatments at 1.7 V_RHE_ in 0.1 M H_2_SO_4_ (a,b) and 0.1 M NaOH (c,d). (a,c) Images recorded immediately after the last oxidation–reduction, (b,d) images recorded after 4 min at 0.05 V_RHE_. The arrows highlight 3D adatom islands that decay in height. All images are 140 × 140 nm^2^. *I*_tip_ = 1 nA, *E*_tip_ = 0.2 V_RHE_.

In acid, the surface presents a high density of small adatom and vacancy islands with a height that in most cases exceeds one monolayer, which is perfectly in line with previous observations.^11^ Please note that it is difficult to discern between adatom and vacancy islands in this 3D morphology, as most of the original terraces have disappeared. Holding the potential at 0.05 V_RHE_ for 4 min does not lead to observable changes of the surface morphology, which indicates that the 3D islands are not mobile in 0.1 M H_2_SO_4_. The reason for this could be the low rate of detachment of adatoms from the island edges as well as their slow diffusion over the terraces, and also a high ES barrier that prevents Au adatom diffusion down onto the lower terraces.^44,45^

The same oxidation–reduction treatment results in a less roughened surface when alkaline electrolyte is used (see Figure 4c). We observe larger terraces with very few 3D adatom islands, which we indicated by white arrows. During the 4 min time at the reduction potential of 0.05 V_RHE_, the 3D islands decay by a removal of the topmost adatom island, which causes a reduction of height and three-dimensionality (see white arrows in Figure 4c,d), while most of the smaller 2D islands disappear. The rapid smoothening of the 3D roughness in 0.1 M NaOH points toward enhanced mobility and a lower ES barrier, as described above for the 2D case. Interestingly, and likely related to this finding, the dissolution rate of gold under anodic oxidation conditions is higher in alkaline compared to acidic media.^3^

Since Au­(111) in 0.1 M NaOH shows very high surface mobility leading to the smoothening and removal of undercoordinated 3D features, it is of utmost interest to study the healing properties of this surface after its roughening during the oxygen evolution reaction (OER), which happens at anodic potentials above 1.7 V_RHE_, i.e., under very oxidative conditions, where immense roughening is expected.^34^ For this, Au(111) was oxidized at 2.45 V for 2 min with current densities of around 10 mA cm^–2^. Subsequently, we reduced the surface at 0.05 V and immediately imaged it with EC-STM (Figure 5a). The resulting surface morphology appears extremely rough, which is attributed to the formation and reduction of a multilayer-thick Au^3+^ oxide, as well as to the enhanced dissolution and redeposition of gold from/at the surface.^3,34^ Intriguingly, even this highly rough surface undergoes a very fast healing process in the alkaline electrolyte (Figure 5b,c).

**Figure 5:**
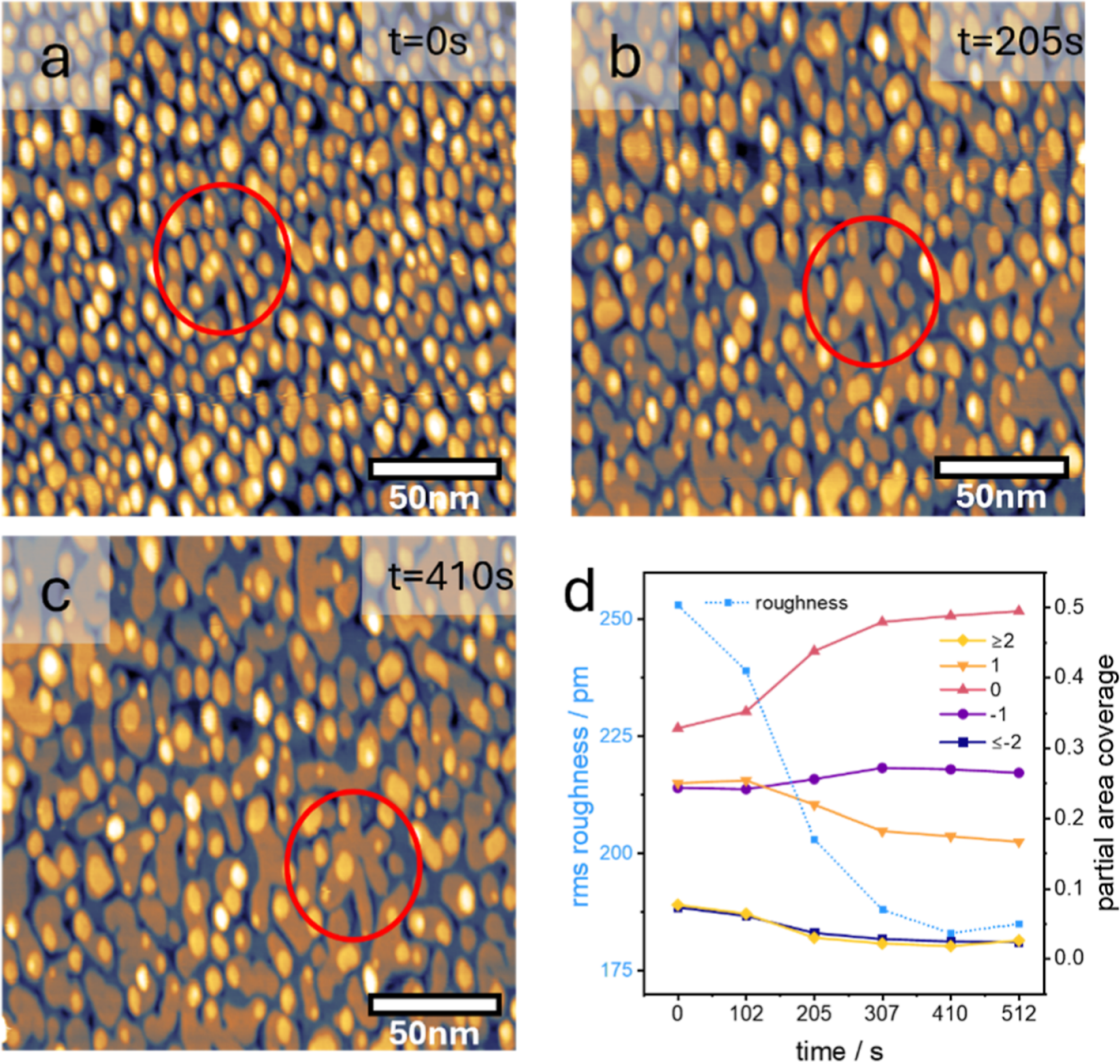
Healing of highly roughened Au(111) after the Oxygen Evolution Reaction (OER). EC-STM images of Au(111) in 0.1 M NaOH after the potential was held at 2.45 V_RHE_ for 2 min and then stepped to *E* = 0.05 V_RHE_. (a) Surface immediately after the reduction step, (b,c) same surface area after holding the potential at 0.05 V_RHE_ for 205 and 410 s. The red circle indicates the same area in the images. (d) Coverage evolution of the central terrace (layer “0”), the upper (“+1”,” ≥ +2”), and lower layers (“–1”, “≤ −2”) with reduction time. Blue dotted line: surface roughness evolution with time. Figure S5 shows additional images. All images are 200 × 200 nm^2^, recorded with *I*_tip_ = 1 nA and *E*_tip_ = 0.2 V_RHE_.

To quantify this smoothening effect, we determined the area (coverage) of each surface layer and its change over time and plotted the results in Figure 5d (details of the image analysis are given in the Supporting Information and Figure S6). The coverage of the central terrace (layer “0”) increases significantly at the expense of both the upper (layer “+1” and layer “≥+2”) and the lower terraces (layers “≤−2”). This suggests that a substantial amount of material diffuses downward from the topmost levels to layer “0”. At the same time, some adatoms landing on layer “0” diffuse into vacancies and fill layers “–1” and “–2”. Consequently, the surface roughness decreases tremendously, as indicated by the dotted blue line in Figure 5d. As noted earlier, this large-scale material transport between layers can only occur if the Ehrlich–Schwoebel (ES) barrier is low, which appears to be the case for Au(111) in the presence of NaOH as electrolyte.

## Conclusion

This EC-STM investigation elucidates the influence of the electrolyte composition on the morphological evolution of the Au(111) surface upon an oxidation–reduction treatment. In both 0.1 M H_2_SO_4_ and 0.1 M NaOH, a single oxidation–reduction treatment induces the formation of monolayer height adatom and vacancy islands. While in the acidic electrolyte these islands remain metastable in number and size within the timeline of the experiments, they rapidly shrink and even disappear in alkaline solution. We attribute these differences to the lower stability of the islands and/or an enhanced surface mobility in 0.1 M NaOH solution compared to 0.1 M H_2_SO_4_.

Repeated oxidation–reduction treatments lead to a 3D roughening of the Au(111) surface, with distinct differences depending on the nature of the electrolyte. At the Au(111)/H_2_SO_4_ interface, the oxidation reduction treatments have high impact on the surface roughness, where small multilayer adatom and vacancy islands are formed. At the Au(111)/NaOH interface, the surface morphology after the treatment is characterized by the presence of larger terraces with only few multilayer adatom and vacancy islands. In alkaline media, island features rapidly decay in height upon reduction; even the 3D multilayer protrusions formed upon polarization in the OER regime flatten through surface healing within less than 10 min. This demonstrates the low island stability and high mobility, visible through faster adatom diffusion, in 0.1 M NaOH. This mobility accelerates the decay and eventual disappearance of the islands by Ostwald and Smoluchowski ripening. Alkaline electrolytes seem to also lower the Ehrlich–Schwoebel barrier for interlayer mass transport, which facilitates the leveling of 3D islands. These findings elucidate the crucial role of electrolyte composition on surface diffusion and restructuring, providing valuable insights for optimizing electrode stability in catalysis.

## Supplementary Material



## Data Availability

The data that support the findings of this study are openly available in InvenioRDM at https://doi.org/10.48323/kmx9g-mpa59

## Notes

The Supporting Information includes further details on the quantification of adatom and vacancy islands as well as additional EC-STM images on the healing process of the roughened Au(111) surface (PDF)
