# Supplementary material for: Dynamics of Adatom and Vacancy Islands on Au(111) in Alkaline and Acidic Media
Source: J Phys Chem C Nanomater Interfaces. 2025 Aug 1;129(32):14411–7. doi: 10.1021/acs.jpcc.5c03661 (PMC12359112; doi:10.1021/acs.jpcc.5c03661)
Supplement: Supplementary file 1 [file jp5c03661_si_001.pdf]

Supporting Information

Dynamics of Adatom and Vacancy Islands on Au(111) in Alkaline and Acidic Media

Toni Moser[1], Francesc Valls Mascaro[1], Julia Kunze-Liebhäuser[1*],

[1]University of Innsbruck, Innrain 52c, 6020 Innsbruck, Austria

(*corresponding author: Julia Kunze-Liebhäuser, email: julia.kunze@uibk.ac.at)

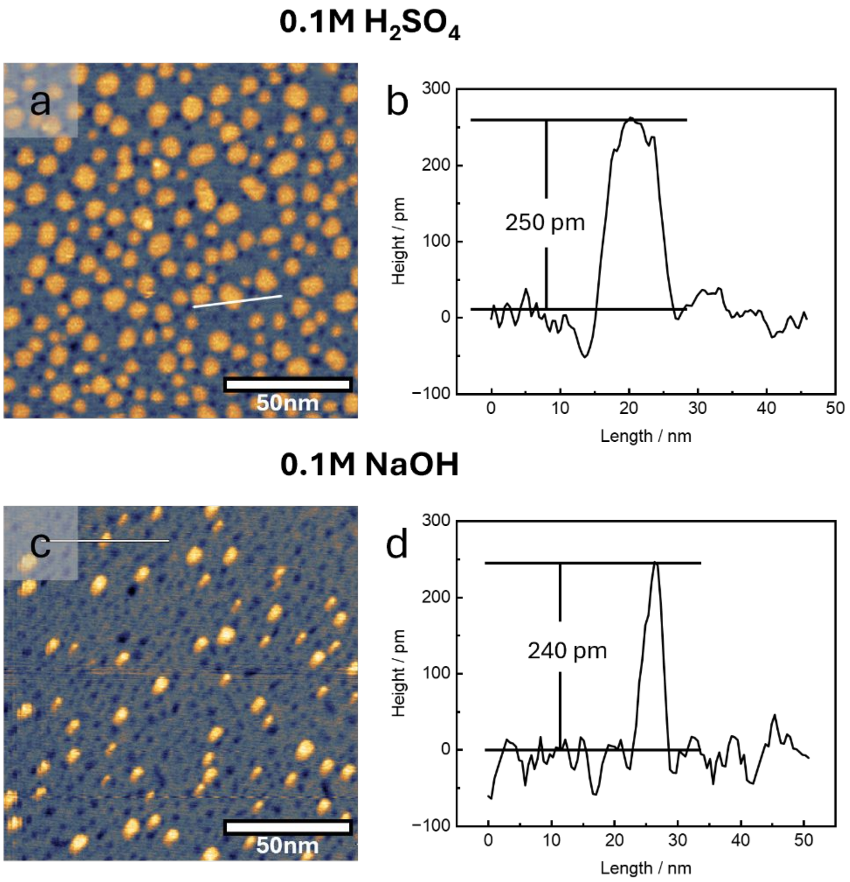

Figure S1. Cross sections of mono-atomic high islands. (a) EC-STM image recorded at E=0.05 V_{RHE} after oxidation at E=1.7 V_{RHE} in 0.1M H_2SO_4, (c) EC-STM image recorded at E=0.05 V_{RHE} after oxidation at E=1.7 V_{RHE} in 0.1M NaOH. The white lines indicate where the cross sections in (a, c) were taken. All EC-STM images are 140 × 140 nm^2.

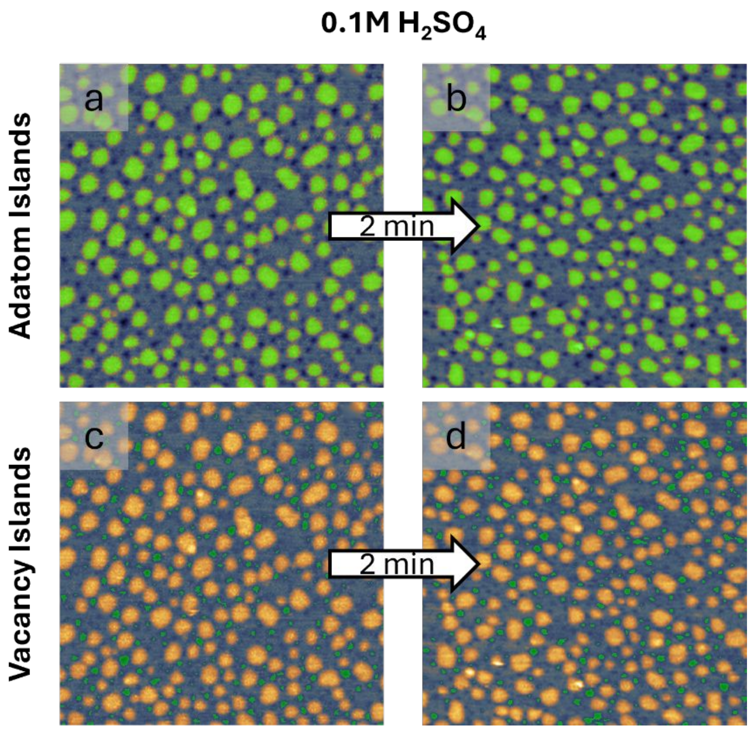

Figure S2. Quantification of adatom and vacancy islands in acid media. EC-STM images recorded at 0.05 V_{RHE} after oxidation at E = 1.7 V_{RHE} in 0.1 M H_2SO_4. (a,b): Adatom islands with heights greater than 100 pm are masked in green. (c,d): Vacancy islands with a depth below -50 pm are masked in green. The quantitative results are presented in Table S1 as well as in Figure 3 of the main paper. All images are 140 × 140 nm².

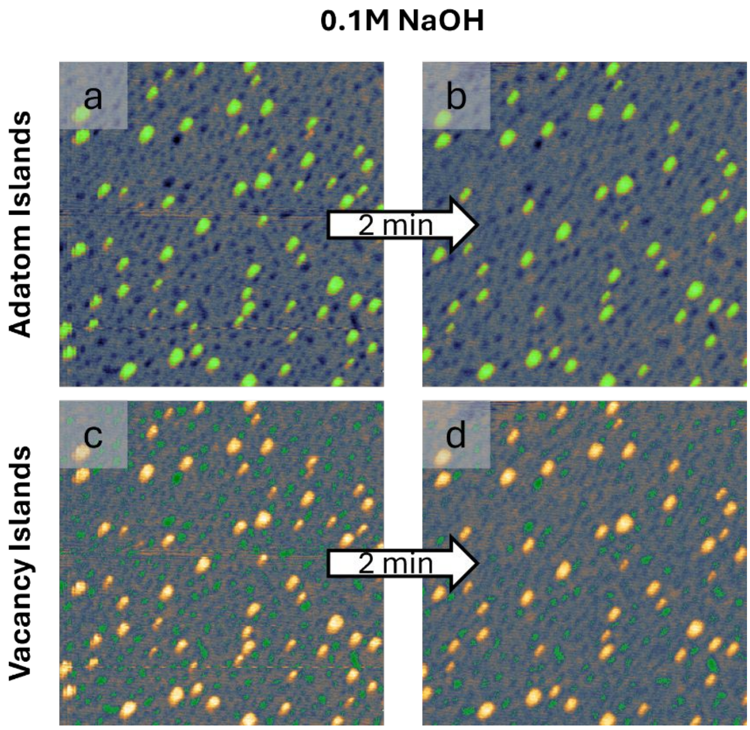

Figure S3. Quantification of adatom and vacancy islands in basic media. EC-STM images recorded at 0.05 V_{RHE} after oxidation at E = 1.7 V_{RHE} in 0.1 M NaOH. (a,b): Adatom islands with heights greater than 100 pm are masked in green. (c,d): Vacancy islands with a depth below -50 pm are masked in green. The quantitative results are presented in Table S2 as well as in Figure 3 of the main paper. All images are 140 × 140 nm².

Table S1. **Summary of the results from Figure S2.** Total area, and counts of adatom and vacancy islands, and percentile change with time ($\frac{x_1-x_0}{x_0}$) from the EC-STM images in Figure S2.

**0.1 M H_2SO_4**	Total area of all adatom islands / nm^2	Number of adatom islands	Total area of all vacancy islands / nm^2	Number of vacancy islands
t=0min	3966	205	680	372
t=2min	3756	201	676	349
Change / %	-5.3	-2.0	-0.6	-6.2

Table S2. Summary of the results from Figure S3. The total area, and counts of adatom and vacancy islands, and percentile change with time ($\frac{x_1-x_0}{x_0}$) from the EC-STM images in Figure S3.

**0.1 M NaOH**	Total area of all adatom islands / nm^2	Number of adatom islands	Total area of all vacancy islands / nm^2	Number of vacancy islands
t=0min	1129	69	1466	530
t=2min	947	56	847.5	313
Change / %	-16.12	-18.9	-42.22	-41.0

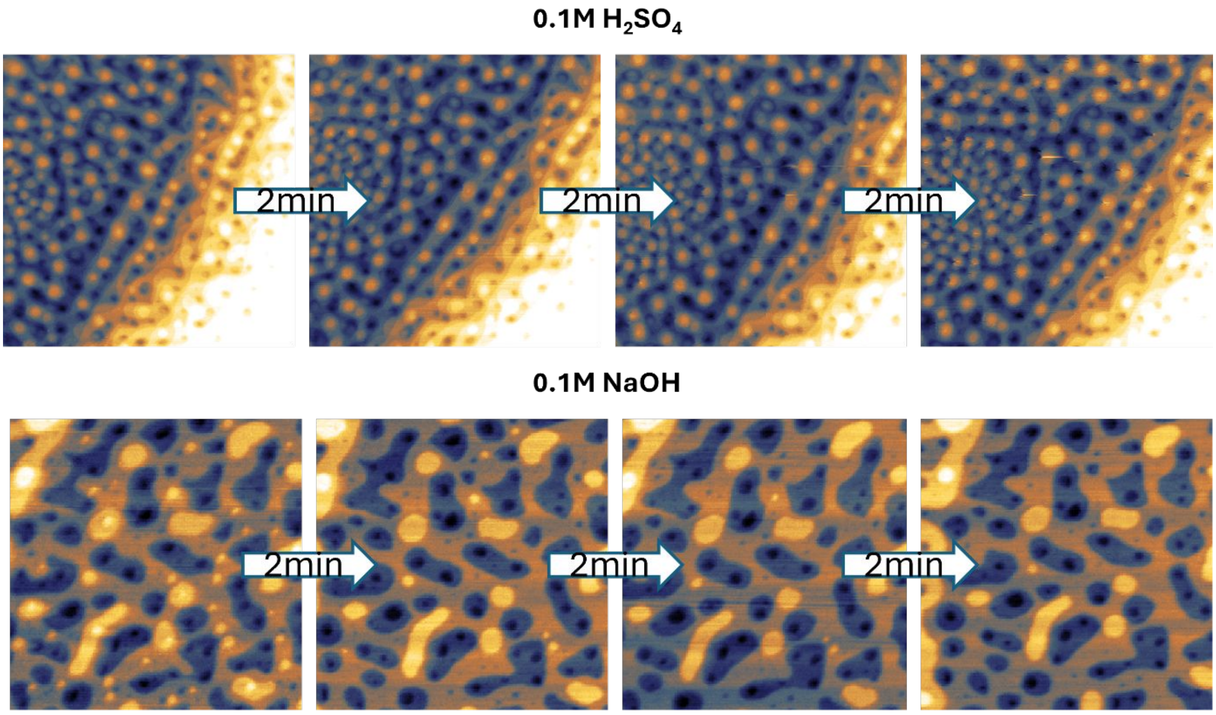

Figure S4. EC-STM images of Au(111) after 10 consecutive oxidation-reduction treatments stepping to 1.7 V_{RHE} and down to 0.05 V in 0.1 M H_2SO_4 (top) and 0.1 M NaOH (bottom). All images are 140 × 140 nm², with I_{tip}=1 nA and E_{tip}=0.2 V_{RHE}.

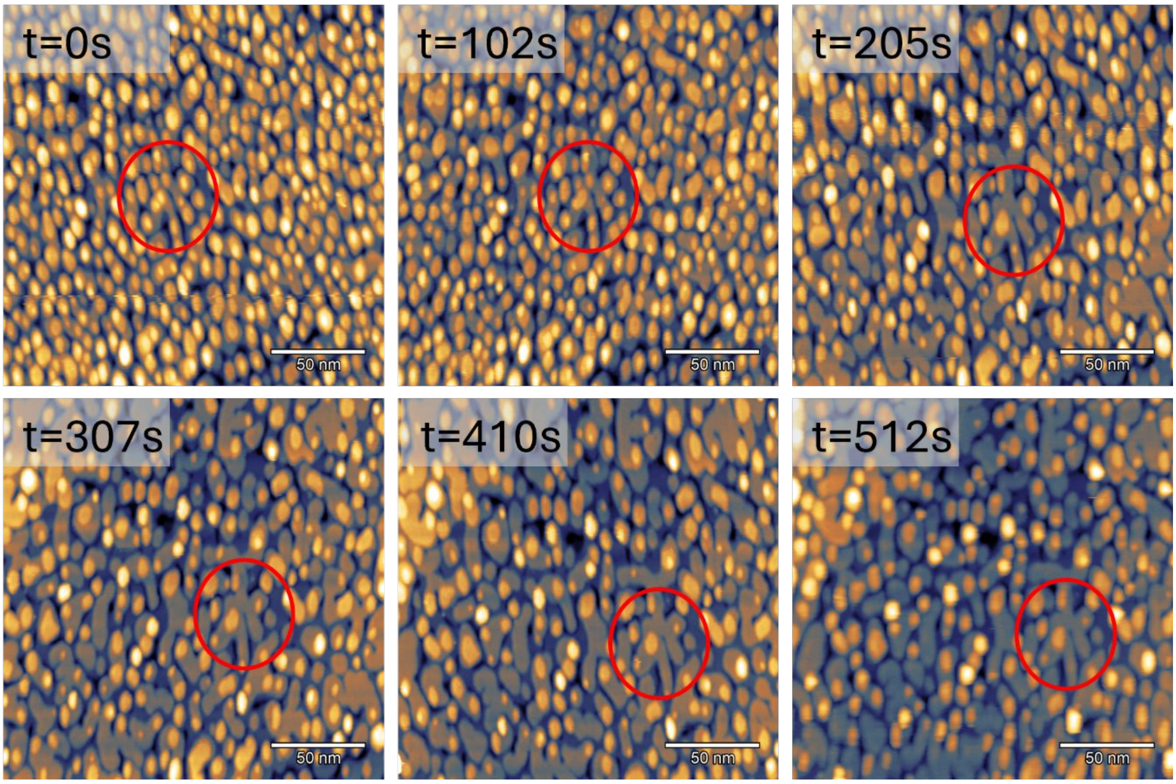

Figure S5. Healing of highly roughened Au(111) surface resulting after the Oxygen Evolution Reaction (OER). Consecutive EC-STM images of Au(111) in 0.1 M NaOH recorded after the potential was held at 2.45 V_{RHE} for 2 minutes and then stepped to E=0.05 V_{RHE}. The red circle indicates the same area in the images. The acquisition time of each image is given at top left corner. All images are 200 × 200 nm², with I_{tip} = 1 nA and E_{tip} = 0.2 V.

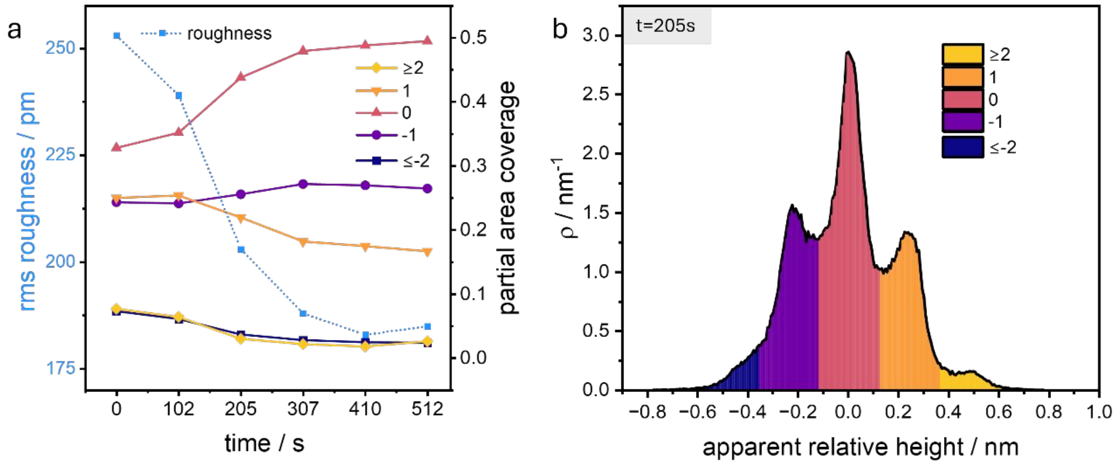

Figure S6. Quantitative evaluation of the surface morphology after the OER. a) Partial coverages of the central terrace (layer "0"), the upper layers ("+1"," ≥+2"), and the lower layers ("-1", "≤-2") versus the time under reduction potential. The blue dotted line shows the roughness evolution of the surface with time. b) Height distribution of the image recorded at t=205 s. The layers -2 to +2 are colored according to the legend.

The STM image analysis shown in Figure S6 was performed by constructing relative height distributions based on the z-values obtained from the images in Figure S5. The peak corresponding to the height of the pristine (original) terrace (referred to as layer “0”) was centered around zero. From there, z-values below –360 pm were assigned to layer “< –2", –360 to –120 pm to layer “–1", –120 to 120 pm to "layer 0", 120 to 360 pm to layer “1", and above 360 pm to layer “>2". The partial surface coverage for each layer can then be quantified.
